# Association between Serum Uric Acid Levels and Perinatal Outcome in Women with Preeclampsia

**DOI:** 10.1155/2021/6611828

**Published:** 2021-04-16

**Authors:** Robinson Uchenna Ugwuanyi, Irozuruike Munachiso Chiege, Felix Eke Agwu, George Uchenna Eleje, Nonso Martin Ifediorah

**Affiliations:** ^1^Department of Obstetrics and Gynaecology, Federal Medical Centre, Umuahia,PMB. 7001, Umuahia, Nigeria; ^2^Effective Care Research Unit, Department of Obstetrics and Gynecology, Nnamdi Azikiwe University, Nnewi Campus, Nnewi, Nigeria; ^3^Department of Obstetrics and Gynecology, Nnamdi Azikiwe University Teaching Hospital, Nnewi,PMB 5025, Nnewi, Nigeria

## Abstract

**Objectives:**

To compare serum uric acid levels with disease severity and perinatal outcome among preeclamptic and normal pregnant women.

**Materials and Methods:**

This was a case-control study carried out in Federal Medical Centre, Umuahia, Nigeria. Consenting pregnant women were consecutively recruited into two groups comprising pregnant women diagnosed with preeclampsia and normotensive nonproteinuric pregnant women. Exclusion criteria included pregnant women who were current smokers, took alcohol, and diagnosed with multiple gestation, diabetes mellitus, or renal failure. Associations between categorical variables such as preeclampsia severity and perinatal outcomes were done using logistic regression while means of continuous variables such as serum uric acid were compared using Student's *t*-test. Data were presented using odds ratios (ORs) with 95% confidence intervals (95% CI) and a statistical significance level set at *P* value ˂ 0.05. Data analysis was done using Statistical Package for Social Sciences version 22.

**Results:**

One hundred and two participants were finally analysed. Fifty-one participants were recruited in each arm. Women with preeclampsia had significantly high serum uric acid level versus controls (6.08 ± 0.49 mg/dL vs. 5.20 ± 0.19; *P* < 0.001). Women with elevated serum uric acid levels (˃6 mg/dL) were found to be 4 times more likely to have severe preeclampsia (*P*=0.022, OR = 4.00, 95% CI = 1.225–13.056), 66 times more likely to have APGAR score ˂7 in the first minute (*P* < 0.001, OR = 66.00, 95% CI = 6.991–623.128), and 3 times more likely to have lower birth weight (*P*=0.038, OR = 3.400, 95% CI = 1.073–10.775) than those with normal serum uric acid levels.

**Conclusions:**

The mean serum uric acid level in a preeclamptic is higher than that of normal pregnant control, and higher levels are associated with severity of the disease and significantly associated with poorer perinatal outcome.

## 1. Introduction

Preeclampsia is a serious pregnancy complication. It is a multisystem disorder characterized by hypertension (blood pressure ≥140/90 mm Hg), proteinuria (24 hr urinary protein ≥0.3 g) with or without pathological edema, beyond the 20th week of gestation in previously normotensive and nonproteinuric woman [1]. Preeclampsia affects approximately 5–7% of all pregnancies and is associated with several complications [2]. The number of babies who die from these disorders is thought to be on the order of 500,000 per annum [3]. Its incidence has continued to increase worldwide accounting for about 60,000 deaths worldwide annually [4, 5]. In developing countries where access to health care is limited, preeclampsia is a leading cause of maternal mortality, causing an estimated >50,000 maternal deaths per year [6]. While maternal death due to preeclampsia is less common in developed countries, maternal morbidity is high and is a major contributor to intensive care unit admissions during pregnancy [7]. Approximately 12 to 25% of fetal growth restriction and small for gestation age infants as well as 15 to 20% of all preterm births are attributable to preeclampsia; the associated complications of prematurity are substantial including neonatal deaths and serious long-term neonatal morbidity [8]. Thus, clinical prediction of disease complications may facilitate initiation of timely management to avert mortality and morbidity in the mother and baby. Although the etiology is still largely unknown, there are a few hypotheses regarding the pathophysiology and prediction of preeclampsia [9–11].

Preeclampsia is associated with uricaemia [12]. Nevertheless, some studies reported that uricaemia is not a consistent predictive factor of preeclampsia [13].

Uric acid is the end product of purine metabolism in humans and is generated by the action of the enzyme, xanthine oxidase, which catalyzes the last two steps of uric acid conversion: hypoxanthine to xanthine and from xanthine to uric acid [14]. There is ample evidence that uric acid has multiple actions impacting on cellular metabolism. Uric acid is a marker of oxidative stress, tissue injury, and renal dysfunction and therefore might be helpful in the prediction of complications of preeclampsia [15, 16]. Elevated uric acid concentrations were first noted in preeclamptic women in the late 1800s [17]. Since then, there have been a lot of conflicting reports in the literature as regards the association between maternal hyperuricaemia and severity of preeclampsia and pregnancy outcome [16, 18–24].

Therefore, measurement of serum uric acid concentration seems to be useful test to predict maternal complications in the management of women with preeclampsia.

This study was designed to determine the role of maternal serum acid in the severity of the disease and perinatal outcome in preeclamptic women and to compare with the normotensive pregnant women (control).

## 2. Materials and Methods

### 2.1. Study Design

This was a case-control study. Pregnant women who correctly completed the consent form were recruited. This study was carried out within a period of six months from 1st November, 2018, to 30th April, 2019.

### 2.2. Study Area

The study was conducted in the antenatal clinic, antenatal, and labor wards of the obstetrics and gynecology department of the Federal Medical Centre, Umuahia, Nigeria. The hospital provides antenatal, delivery, postnatal, and family planning services and serves as a referral centre for both private and government health institutions in the state and its environment.

### 2.3. Study Population

Pregnant women attending antenatal clinic, as well as those admitted into the antenatal and labor wards, who voluntarily consented to participate following detailed information on the nature and purpose of the study, were recruited. Group A comprised pregnant women diagnosed with preeclampsia after 20 weeks' gestational age while group B comprised normal pregnant women after 20 weeks' gestational age. Participants` confidentiality was ensured.

### 2.4. Diagnostic Criteria for Preeclampsia

The diagnosis of preeclampsia was made using blood pressure measurements at least 4 hours apart after 20 weeks' gestation in which the systolic blood pressure was ≥140 mmHg and/or diastolic blood pressure is ≥90 mmHg (using Korotkoff V), plus significant proteinuria of ≥1+ on dip stick assessment. The blood pressure measurement was done using a mercury sphygmomanometer (Accoson, England) and proteinuria was determined using self-stik+2 (Chungdo Pharmaceutical Company Limited, Korea). Preeclampsia was classified as mild when the systolic blood pressure is ≥140 mmHg but <160 mmHg and/or a diastolic blood pressure of ≥90 mmHg but <110 mmHg with significant proteinuria. It was classified as severe when a single systolic blood pressure measurement is ≥160 mmHg and/or diastolic blood pressure of ≥110 mmHg with significant proteinuria, and/or massive proteinuria on dipstick of ≥3+.

### 2.5. Sample Size Determination

The sample size was calculated by using the prevalence of high serum uric acid levels in preeclamptics in two independent studies done in Benin, South Nigeria [25] and Tehran in Iran [26]. The formula, used for calculating the sample size for a case-control study, was as follows [27]:

(1) $$\begin{array}{c} \frac{n}{r}=\frac{r+1\left(P^{x}\right)\left(1-P^{x}\right){\left(Z\beta +Z\alpha_{2}\right)}^{2}}{{\left(P_{1}-P_{2}\right)}^{2}}, \end{array}$$

where *n* = minimum sample size, *r* = ratio of control to cases which is 1 for equal number of cases and control, *P*^*x*^ = average proportion exposed = proportion of exposed cases + proportion of control exposed/2, Z*β* = standard normal variate for level at 95% confidence interval = 1.96, Z*α*_2_ = standard normal variate at statistical power of 90% = 1.28, *P*_1_ − *P*_2_  = effect size or different in proportion expected based on previous studies, *P*_1_ is proportion in cases and *P*_2_ is proportion in control, *P*_1_ = 0.5 (proportion of preeclamptic with hyperuricaemia in Benin study) [25], and *P*_2_ = 0.254 (proportion of normotensives with hyperuricaemia in Tehran study) [26]:

(2) $$\begin{array}{c} P^{x}=0.377,\\ N=\frac{0.377\times 0.623\times 10.4976}{0.060516}=40.7426. \end{array}$$

This was approximately 41.

The response rate of 80% became

(3) $$\begin{array}{c} n=\frac{n}{1-r},\\ \frac{41}{1-0.2}=\frac{41}{0.8}=51.25, \end{array}$$

which was approximately 51 on each arm making overall sample size of 102.

### 2.6. Inclusion Criteria

Pregnant women diagnosed with preeclampsia and normal pregnant women at 20 weeks' gestational age and above were included.

### 2.7. Exclusion Criteria

Pregnant women who are current smokers, taking alcohol, and those with of multiple gestations, diabetes mellitus, thyroid disorder, and renal failure were excluded.

### 2.8. Recruitment of Subjects

The participants were recruited from the antenatal clinic, antenatal, and labor wards of the Federal Medical Centre, Umuahia, Nigeria.

### 2.9. Recruitment of Cases

Every preeclamptic pregnant woman who met the eligibility criteria and was willing to participate in the study was recruited. Participants in the study were recruited consecutively until the required sample size was complete.

### 2.10. Recruitment of Control

For each case recruited, a normotensive, nonproteinuric pregnant woman who met the eligibility criteria and was willing to participate in the study was consecutively recruited as control. Controls were matched for age, parity, and gestational age. Gestational age was calculated by dates (from last normal menstrual period) and ultrasound measurements in first half of pregnancy. All subjects included in the study were recorded on a specially designed proforma.

### 2.11. Data Collection Tool

A semistructured study proforma was used for data collection. The information recorded included sociodemographic data, as well as height, weight, and blood pressure which were measured and documented. The result of the urinalysis was also documented.

### 2.12. Anthropometry

The heights of the women were measured and weight determined. These measurements were done using a weighing scale with incorporated stadiometer PyrochyRGZ-160 (Pyrochy Medical, England) with error margins of 0.5 cm and 0.5 kg, respectively. Body mass index was calculated as weight (kg) divided by (height)^2^ (m^2^). Effort was made to match the control by anthropometry.

### 2.13. Blood Sample Collection

About 5 ml of blood was drawn from the antecubital vein using a sterile needle and syringe into a sterile plain tube. The sample in the plain tube was allowed to stand for 30 minutes undisturbed so as to clot. Serum was separated by centrifugation for 10 minutes at 3000 revolutions per second. The serum was then aspirated into plain bottles and stored at minus 20°C until time of analysis which was done in batches within 2 weeks of sample collection.

### 2.14. Procedure

The analysis of serum uric was performed by a senior Medical Laboratory Scientist under the supervision of a chemical pathologist at the Federal Medical Centre, Umuahia, Nigeria. The assay was done using commercially manufactured ready for use kit by Randox Laboratories Limited, 55 Diamond Road, Crumlin, County Antrim, BT29 4QY, United Kingdom.

## 3. Ethical Consideration

### 3.1. Ethical Approval

This study was approved by the Health Research and Ethical Committee of the Federal Medical Centre, Umuahia, Nigeria (approval number: FMC/QEH/G.596/Vol.10/397; approval date: 4 July 2017). It was carried out according to the national and international regulations governing the use of human subjects in biomedical research.

### 3.2. Data Analysis

Data analysis was done using Statistical Package for Social Sciences (SPSS) version 22. Descriptive statistics which include frequency and percentages were used to summarize categorical variables while means and standard deviations were obtained for continuous variables. Associations between categorical variables such as preeclampsia severity and pregnancy outcomes were done using logistic regression and chi-square while means of continuous variables such as serum uric acid were compared using Student's *t*-test. Data were presented using odds ratios (ORs) with 95% confidence intervals (95% CI). The level of statistical significance was set at *P* value of less than 0.05. Results are presented in tables and charts.

## 4. Results

**Figure 1:**
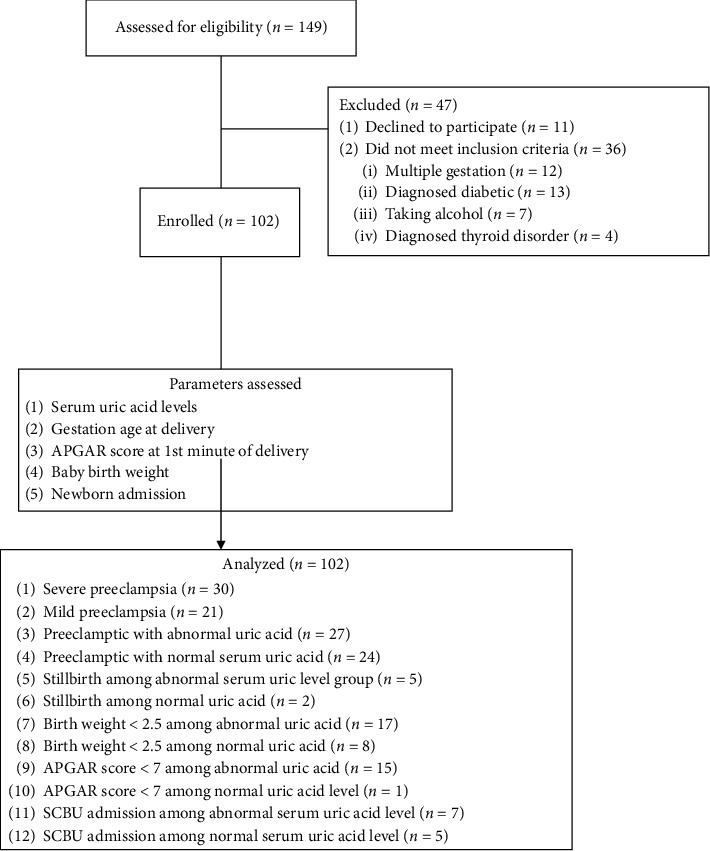
Flow pattern of serum uric acid level and pregnancy outcome among preeclamptic normal pregnant women. SCBU = special care baby unit.

One hundred and forty-nine participants were initially approached and screened for inclusion in the study, following which 31.54% of the participants were excluded while one hundred and two participants were finally recruited. The flowchart of the participants is shown in Figure 1. They were categorized into two groups: group A which comprised 51 pregnant women diagnosed with preeclampsia and group B which comprised 51 normotensive, nonproteinuric controls.

The mean maternal age and gestational ages of both subjects and control are presented in Table 1. The mean ages in both cases and control were 30.96 ± 4.36 and 29.86 ± 4.97, respectively, while the mean gestational ages in both wings were 36.92 ± 4.72 and 38.65 ± 1.65, respectively.  Table 2 shows the parity and booking status of the participants which showed an almost equal distribution across the two arms. The pregnancy outcome of the subjects is shown in Table 3 which shows that most (52.9%) of those diagnosed with preeclampsia had caesarean section. Table 3 also reveals that 19.6% of those diagnosed with preeclampsia had preterm delivery as against the 3.9% of the nonpreeclamptic group.

Table 4 shows the comparison between the mean arterial blood pressure between the preeclamptics (124.86 ± 11.99 mmHg) and nonpreeclamptics (84.18 ± 6.79 mmHg) which was statistically significant (*P* < 0.001). Figure 1 shows that 59% of those diagnosed with preeclampsia had severe preeclampsia as against 41% that had mild preeclampsia.

Women with preeclampsia had significantly high serum uric acid level compared to controls (6.08 ± 0.49 mg/dL vs 5.20 ± 0.19; *P* < 0.001).

Proportion of the preeclamptics that had abnormal uric acid level (>6 mg/dL) as shown on Figure 1 was 53% while 47% had normal serum uric acid level (≤6 mg/dL).

Table 5 shows a significant association between serum uric acid levels and severity of preeclampsia (*P*=0.022) where women with abnormal serum uric acid levels (>6 mg/dL) were found to be 4 times more likely to have severe preeclampsia than those with normal serum uric acid level.

The association between serum uric acid levels and pregnancy outcome is presented in Table 6 which shows that preeclamptic women with abnormal serum uric acid levels (>6 mg/dL) were 66 times more likely to have APGAR score <7 in the first minute (*P* < 0.001, OR = 66.00, 95% CI = 6.991–623.128) and also 3 times more likely to have low birth weight than those with normal serum uric acid (*P*=0.038, OR = 3.400, 95% CI = 1.073–10.775).

Up to 25.9% of the preeclamptics that were hyperuricaemic (>6 mg/dL) had special care baby unit admission as against 20.8% of those with normal serum uric acid level. However, this was statistically insignificant (*P* > 0.005). Up to 20.8% of those preeclamptics with abnormal serum uric acid had stillbirth as against 7.9% of the preeclamptics that had normal serum uric acid level.

Our findings also showed that 59.0% of those that were diagnosed with preeclampsia had severe preeclampsia and that 53.0% of the preeclamptics had abnormal uric acid (>6 mg/dL).

## 5. Discussion

The mean serum uric acid level in the preeclamptics and nonpreeclamptics in this study was 6.08 ± 0.49 and 5.20 ± 0.19, respectively. This shows that preeclamptic women had significantly higher uric acid levels when compared to the normotensive control (*t* = 11.871, *P* < 0.001). Similar findings were noted in other studies done elsewhere [16, 18, 28–31]. This could be due to the postulation that hypoxia and ischemia of the placenta and cytokines such as interferon induce the expression of xanthine oxidase and therefore increase the production of uric acid and also reactive oxygen species in preeclamptics. It has also been postulated that reduced uric acid clearance secondary to reduced glomerular filtration rate, increased reabsorption, and decreased secretion may be the reasons for elevated serum uric acid levels in women with preeclampsia [20].

There was a significant association between serum uric acid levels and severity of preeclampsia (*P*=0.022) in our study where it was found that women with abnormal uric acid level (˃6 mg/dL) were 4 times more likely to have severe preeclampsia than those with normal serum uric acid level (≤6 mg/dL). This was in keeping with the result of similar study done by Toshniwal and Lamba in Gujirat, India, where uric acid was suggested to be a good marker of severity of preeclampsia [12]. This can be explained by the fact that uric acid, being a potent mediator of inflammation, promotes endothelial dysfunction per se which promotes hypertension, vascular disease, and renal disease. Similar studies done by Punthumapol and Kittichotpanich in Bangkok and Osakwe et al. in Nnewi, Southeast Nigeria, showed similar results to our study [28, 29]. Our study revealed that 53% of patients who have severe preeclampsia have abnormal serum uric acid level (˃6 mg/dL) which is in keeping with a study done in Benin, South Nigeria, where 50% of patient diagnosed with severe preeclamptic had abnormal serum uric acid [25]. Though our sample size of 30 severe preeclamptics appeared smaller that the sample size of 40 severe preeclamptic recruited in Benin study, the homogeneous population and gestational age matched control in our study ensured the reliability of the results. Our result has buttressed the fact that there is an association between severe preeclampsia and elevated serum uric acid.

Our study also looked at the association between serum uric acid and pregnancy outcome among the preeclamptics where it was found that 75% of the neonates of preeclamptic mothers who had abnormal serum uric acid (˃6 mg/dL) had a 1^st^ minute APGAR score of ˂7 as against 4.3% of the neonates of preeclamptic mothers who had normal serum uric acid level (≤6 mg/dL) (*P* < 0.001, OR = 66.00, 95% C. I = 6.991–623.128) which was statistically significant. It was also discovered that 63.0% of neonates of the hyperuricemic preeclamptics had low birth weight as compared to 33.3% of neonates of the normouricemic preeclamptics (*P*=0.038, OR = 3.400, 95% C. I = 1.073–10.775). This was also statistically significant. This is in keeping with the findings in a similar study done by other authors [19, 32], where they reported significant increased number of low birth weight in babies born to hyperuricaemic preeclamptic in comparison with babies born to normouricemic preeclamptic mothers. Other authors also recorded similar experience [16, 18]. This indicated that raised uric acid probably causes reduced placental function which may result in low birth weight. Hussaine and Sonogara have independently recommended that lowering serum uric acid levels in preeclamptic and prompt intervention can improve pregnancy outcome in preeclamptics [33]. Similarly, Cicero et al. reported significantly associated negative maternal outcomes in women with preeclampsia and higher serum uric acid level, while antihypertensive treatment, number of previous deliveries, and blood pressure control at deliveries appeared to be protective. Cicero et al. further reported significantly negative fetal outcomes in women with preeclampsia while antihypertensive treatment and blood pressure control at delivery seemed to be protective [34].

In our study, there were more stillbirths in neonates of hyperuricaemic preeclamptic mothers than the normouricemic preeclamptic mothers; however, this was not statistically significant (*P*=0.181, OR = 3.289, 95% C. I = 0.575–18.834). This was in keeping with the result of similar study done by Hussain et al. The small sample size may have been responsible for the nonsignificant difference noted in the two groups. In our study, 25.9% of babies born to hyperuricaemic preeclamptic mothers were admitted to special care baby unit as against 20.8% of those born to normouricemic preeclamptic mothers. However, this was not statistically significant which could be explained by the small sample size.

In our study, there was no significant difference in the subjects on both arms in terms of age, level of education, marital status, booking status, and parity. This added credence to this study. Our findings are similar to the work done by Razia et al. where they found no significant difference between the two arms in terms of maternal age [21]. However, there was statistically significant difference between the two arms in terms of mode of delivery and gestational age at delivery. Up to 52.9% of the preeclamptics were delivered via caesarean section as against the 19.6% of the control whereas 19.6% of the preeclamptics were delivered preterm as against 3.9% of the control. This finding differed from Razia et al. study which found no significant difference in gestational age between the two groups [21].

The strength of this study was that we relied on the fact that all cases that met the inclusion criteria were included. However, the study may be limited by lack of standardized normal values of uric acid for different gestational ages in the study environment. Another limitation of the study is that serum uric acid was considered as single variable without considering relevant covariate as age, renal function, and body mass index.

## 6. Conclusion

The mean serum uric acid level in a preeclamptic is higher than that of normotensive, nonproteinuric women. Serum uric acid levels in patients with preeclampsia are associated with severity of the disease. There is significant association between serum uric acid levels in preeclamptics with perinatal outcome. Significant increased number of low-birth-weight fetuses was observed in babies born to hyperuricaemic preeclamptic mothers in comparison with babies born to normouricemic preeclamptic mothers.

## Figures and Tables

**Table 1:** Mean maternal age and gestational age of subjects.

	Preeclamptic	Non preeclamptic	*T*	*P* value
	Mean ± SD	Mean ± SD		
Age	30.96 ± 4.38	29.86 ± 4.97	1.184	0.239
GA	36.92 ± 4.72	38.65 ± 1.65	2.467	0.015

**Table 2:** Parities and booking status of subjects.

	Preeclamptic *n* (%)	Nonpreeclamptic *n* (%)	*X* ^2^	*P* value
Parity				
0	32 (62.7)	32 (62.7)	0.000	1.000
1–4	18 (35.3)	18 (35.3)		
>4	1 (2.0)	1 (2.0)		

Booking status				
Booked	27 (52.9)	36 (70.6)	3.363	0.067
Unbooked	24 (47.1)	15 (29.4)		

**Table 3:** Pregnancy outcome of the subjects.

	Preeclamptic *n* (%)	Non preeclamptic *n* (%)	*χ*2	*P* value
*SCBU admission*	12 (23.5)	8 (15.7)	0.995	0.318
*No SCBU admission*	39 (76.5)	43 (84.3)		
*Mode of delivery*				
C/S	27 (52.9)	10 (19.6)	12.257	<0.001
SVD	24 (47.1)	41 (80.4)		
*Live birth*	44 (86.3)	50 (98.0)	4.883	0.027
*Still birth*	7 (13.7)	1 (2.0)		
Preterm	10 (19.6)	2 (3.9)	6.044	0.014
Term	41 (80.4)	49 (96.1)		

SCBU=special care baby unit. C/S= caesarean section. SVD=spontaneous vaginal delivery.

**Table 4:** Comparison of mean blood pressure between preeclamptics and nonpreeclamptics

	Preeclamptic mean ± SD	Nonpreeclamptic mean ± SD	*T*	*P* value
SBP (mmHg)	16.7.45 ± 17.75	114.61 ± 14.29	16.559	<0.001
DBP (mmHg)	101.43 ± 17.61	65.59 ± 22.13	9.050	<0.001
MABP(mmHg)	124.86 ± 11.99	84.18 ± 6.79	21.083	<0.001

**Table 5:** Association between serum uric acid levels and severity of preeclampsia.

Preeclampsia	Serum uric acid		OR	95% CI for OR	*P* value
	>6 mg/dL *n* (%)	≤6 mg/dL *n* (%)			
Severe	20 (74.1)	10 (41.7)	4.000	1.225–13.056	0.022
Mild	7 (25.9)	14 (58.3)			

**Table 6:** Association between serum uric acid levels and pregnancy outcomes.

	Serum uric acid		OR	95% CI for OR	*P* value
	>6 mg/dL *n* (%)	≤6 mg/dL *n* (%)			
*SCBU admission*	7 (25.9)	5 (20.8)	1.330	0.360–4.920	0.669
*No SCBU admission*	20 (74.1)	19 (79.2)			
*APGAR score*					
<7	15 (75.0)	1 (4.3)	66.000	6.991–623.128	<0.001
≥7	5 (25.0)	22 (95.7)			
*Birth weight (kg)*					
<2.5	17 (63.0)	8 (33.3)	3.400	1.073–10.775	0.038
≥2.5	10 (37.0)	16 (66.7)			
*Live birth*	19 (79.2)	25 (92.6)	3.289	0.575–18.834	0.181
*Still birth*	5(20.8)	2 (7.4)			

## Data Availability

The data used to support the findings of this study are available from the corresponding author upon request.
